# Rheological Properties of Emulsions Stabilized by Cellulose Derivatives with the Addition of Ethyl Alcohol

**DOI:** 10.3390/ma17246090

**Published:** 2024-12-13

**Authors:** Sylwia Różańska, Jacek Różański, Patrycja Wagner, Ewelina Warmbier-Wytykowska

**Affiliations:** Division of Chemical Engineering and Equipment, Faculty of Chemical Technology, Poznan University of Technology, ul. Berdychowo 4, 60-965 Poznan, Poland; jacek.rozanski@put.poznan.pl (J.R.); patrycja.wagner@put.poznan.pl (P.W.); ewelina.warmbier@doctorate.put.poznan.pl (E.W.-W.)

**Keywords:** emulsions, cellulose derivatives, emulsions stability, viscoelsticity

## Abstract

The paper presents the results of research on the rheological properties and stability of oil-in-water emulsions containing cellulose derivatives: methylcellulose, hydroxyethylcellulose, and hydroxypropylmethylcellulose. The continuous phase of the emulsion was a 70% ethanol (EtOH) solution by volume. The dispersed phase consisted of mineral, linseed, and canola oils (20% by volume). Rheological measurements were performed in both steady and oscillatory flow. Emulsion stability was assessed on visual observation and changes in droplet diameter over a period of 5 months after preparation. Relatively stable emulsions were obtained without the addition of low-molecular-weight surfactants, exhibiting viscoelastic properties. The presence of ethanol in the continuous phase significantly slowed down the processes of emulsion sedimentation or creaming, as well as droplet coalescence. The reasons for the slow phase separation were linked to changes in density and zero-shear viscosity of the continuous phase caused by the addition of EtOH. All emulsions were highly polydisperse, and the addition of methylcellulose and hydroxypropylmethylcellulose further led to the formation of strongly flocculated emulsions. Droplet flocculation resulted in highly viscoelastic fluids. In particular, for emulsions containing hydroxypropylmethylcellulose, the ratio of the storage modulus to the loss modulus approached a value close to 0.1, which is characteristic of gels.

## 1. Introduction

Cellulose is the most common polysaccharide found in nature. It is a linear polymer composed of (1–4) linked β-D-glucopyranosyl units, which create a rigid structure due to the presence of intramolecular hydrogen bonds [1]. Cellulose is insoluble in cold and hot water and in organic solvents, which limits its range of applications. For this reason, cellulose is modified with a view to obtaining water-soluble polymers. From a practical perspective, the most important cellulose ethers include carboxymethylcellulose sodium salt (Na-CMC), methylcellulose (MC), hydroxyethylcellulose (HEC), hydroxypropylcellulose (HPC), and hydroxypropylmethylcellulose (HPMC).

Cellulose derivatives are frequently utilized in the production of hydrogels. Hydrogels based on these compounds are biocompatible; hence, they are used in biomedical engineering as components of drug delivery matrices (carriers for the controlled release of active substances in medicines) [2,3,4,5], cellular immobilization [6], diagnostics [7], and separation of biomolecules or cells [8]. During the COVID-19 pandemic, cellulose-based thickeners and gelling agents were employed as ingredients in alcohol-based hand sanitizers (ABHRs) [9,10]. Berardi et al. [9] demonstrated that a majority of commercially available hand sanitizers contain carbomers and cellulose derivatives. Solutions of cellulose derivatives with added ethyl alcohol are also utilized in the food and cosmetics industries [11], while emulsions containing ethanol in the aqueous phase are employed in the production of alcoholic beverages and quick-drying water-soluble paints [12,13].

The rheological properties of aqueous solutions of hydrocolloids, including cellulose derivatives, are well-documented in the literature [14,15,16,17,18]. However, there is limited research on how the properties of solutions of these compounds change when alcohol is added [10,19,20]. Roberts et al. [20] studied the influence of alcohol on the rate and mechanisms of release of aspirin from HPMC hydrophilic matrices containing up to 40% ethyl alcohol. Parinelli et al. [10] investigated how varying concentrations of ethanol affected the properties of HPMC, HEC, and Na-CMC solutions with different molecular weights. The viscosity of the solutions of all the polymers increased with alcohol concentrations up to about 40% wt. and decreased at higher ethanol levels. Ethanol had a more pronounced effect on the viscosity of solutions containing lower molecular weight polymers.

The influence of ethanol on the rheological and structural properties of κ-carrageenan solutions was studied by Yang et al. [21]. In that case, the addition of ethanol led to the formation of strong gels. The rheology and microstructural results suggested that the inclusion of ethanol up to 20% wt. facilitated κ-carrageenan gel formation and improved gel strength by increasing the κ-carrageenan junction size and numbers.

Brunchi et al. [22] examined how the addition of ethanol affected the properties of xanthan gum. The results indicate that the conformation of macromolecular chains changes with varying XG concentrations and the volume fraction of added EtOH, promoting their aggregation. Furthermore, when EtOH content exceeds 70% vol., this effect leads to phase separation.

Emulsions are inherently thermodynamically unstable multiphase systems. Ensuring appropriate stability of emulsions, as well as suspensions and composites, is crucial from the perspective of the practical application of such materials [23,24]. To slow down processes such as coalescence, creaming, or sedimentation of droplets, emulsifiers are incorporated into the emulsion [25]. The stability of emulsions can be enhanced by incorporating compounds into the continuous phase that adsorb at the interface, as well as by modifying the densities and viscosities of the continuous phase. A commonly employed method for increasing the viscosity of the aqueous phase involves dissolving polymers within it. In emulsions used in the food, cosmetics, and pharmaceutical industries, the most commonly utilized polysaccharides include guar gum, xanthan gum, gum arabic, and various cellulose derivatives [10]. At the same time, adding ethyl alcohol to the aqueous phase of the emulsion can modify its density and interfacial tension, thereby changing emulsion stability. There is a notable lack of systematic studies in the literature on emulsions containing alcohol that are stabilized by small molecular surfactants or polymers. Consequently, information regarding these systems remains limited [26,27,28,29,30].

Dickinson and Stainsby [31] described ethanol/water solutions as thermodynamically non-ideal mixtures due to the nonlinear changes in their physical properties with increasing ethanol concentrations. Thus, it is essential to investigate the impact of alcohol addition on the stability and characteristics of emulsions [25,28]. In emulsions with a continuous phase containing ethanol at high concentrations, a greater solubility of oil in the aqueous phase was noted, leading to an accelerated rate of emulsion maturation. Consequently, the phenomenon of Ostwald ripening is expected to play a significant role in this context [32,33]

Attempts to develop a stable emulsion system were undertaken by Xu et al. [34]. The resulting stable E/O emulsion system contained 95% ethanol as the dispersed phase, sunflower oil as the continuous phase, and a nonionic surfactant MO-750 as the emulsifying agent. The ethanol content significantly influences the stability of oil–ethanol emulsions, likely by affecting the formation of the interfacial film.

Ferreira et al. [28] demonstrated that ethanol in the aqueous phase significantly reduced emulsion droplet size, reaching a minimum at an ethanol concentration of 40%. For the three surfactants tested (Tween 20, Tween 80, and Lecithin), the droplet diameters at 40% ethanol content were comparable, suggesting that close to this concentration of ethanol may be present in the surfactant monolayer, leading to significant changes in interfacial tension. At ethanol concentrations exceeding 40%, all emulsions became unstable, and the interfacial tension matched that of samples containing only ethanol. This observation indicates that the surfactants were effectively displaced from the interface. However, at ethanol concentrations below 40%, the emulsions remained stable for up to 10 months without any change in droplet size.

In their study, Xi et al. [35] analyzed the rheological properties, microstructure, and texture of emulsion gels containing alcohol, depending on the concentration of the dispersed phase. The authors showed that the properties of ethanol-induced whey protein emulsion gels depended strongly on the oil content. Increasing the amount of oil led to an increased structural strength, brittleness, and water-holding capacity of the emulsion gels while also reducing gelation time.

Medina-Torres et al. [26] investigated the properties of emulsions with the addition of ethyl alcohol and different caseinates. They showed that the addition of alcohol increased the stability of the emulsions because ethanol causes a significant reduction in the interfacial tension between the oil and the continuum phase containing the protein in the solution [36]. Thus, homogenizing a premixed emulsion containing alcohol produces oil droplets with a significantly smaller average size (i.e., <5 m) than the equivalent alcohol-free systems [37].

The aim of the study presented in this paper was to develop emulsions stabilized with three cellulose derivatives: hydroxyethylcellulose (HEC), methylcellulose (MC), and hydroxypropylomethylcellulose (HPMC) dissolved in ethyl alcohol solutions at concentrations of 70% vol. The dispersed phase was composed of mineral, linseed, and rapeseed oils (20% by volume). For comparison, tests were also conducted on emulsions that did not contain ethyl alcohol. The emulsions developed in this study have the potential for use in cosmetic formulations and as drug carriers, owing to their biocompatibility and environmental sustainability.

## 2. Materials and Methods

### 2.1. Materials

The emulsions investigated in this study were stabilized with following hydrocolloids: hydroxypropylmethylcellulose (HPMC, Konimpex, Sp. z. o. o., Konin, Poland), hydroxyethylcellulose (HEC, Merck, Darmstadt, Germany), and methylcellulose (MC A40M, Dow Chemical Company, Midland, MI, USA). The concentrations of HEC and MC were 0.5, 0.8, 1.5, and 2% wt., respectively, while the concentrations of HPMC were 0.3, 0.5, and 0.7% wt., respectively. The hydrocolloid concentrations given in the study refer to the continuous phase of the emulsion, which consisted of distilled water mixed with ethanol at a concentration of 70% vol. (Wyborowa S.A. Poznań, Poznań, Poland). The aforementioned hydrocolloids were chosen due to their good solubility in aqueous ethanol solutions [10]. The dispersed phase consisted of three types of oils: linseed oil (LO), canola oil (CO) (Bunge Poland, Warsaw, Poland), and mineral oil (MO) (Institute of Petroleum Technology, Krakow, Poland). The concentration of the dispersed phase in all analyzed emulsions was 20% vol. The applied concentrations of the individual compounds are presented in Table 1. The choice of linseed oil and canola oil for the study stems from the use of emulsions containing these oils in the food, pharmaceutical, and cosmetics industries. Naturally derived oils, in addition to triglycerides, may contain a wide range of other compounds, including small amounts of natural surfactants. For these reasons, the study also included mineral oil, which consists solely of a mixture of liquid hydrocarbons.

### 2.2. Preparations of Solutions and Emulsions

In order to obtain aqueous polymer/ethanol solutions at specific percentage concentrations (% wt.), the polymers were gradually added to a beaker containing a measured amount of water/ethanol mixture. Solutions were stirred for approximately 24 h. After this time, the solutions were placed in a refrigerator and stored at 4 °C.

To prepare an emulsion with a dispersed phase concentration of 20% vol., appropriate amounts of the polymer/ethanol mixture and oil were taken and mixed using an IKA Turrax T18 rotor-stator homogenizer (IKA, Werke GmbH & Co., Mindelheim, Germany) for 5 min at a speed of 8000 rpm. All emulsions were produced under the same conditions.

### 2.3. Optical Microscopy

The optical micrographs of the emulsions were captured by a Nikon Eclipse 50i microscope (Tokyo, Japan). The images of the droplets were captured through the CCD camera (OptaTech, Espoo, Finland) mounted on optical microscopy. A drop of emulsion was placed between a microscope slide and cover slip. The captured images were analyzed using the MATLAB program, version R2023a (MathWorks, Inc., Natick, MA, USA). The average number of droplets used for calculations was 20,000 ± 50.

Table 2 and Table 3 summarize the value of Sauter mean diameter (*d*_32_) and polydispersity index (PDI) of the droplets calculated based on the equations [38,39].

(1) $$d_{32}=\frac{\overset{N}{\underset{i=1}{\Sigma }}n_{i}d_{i}^{3}}{\overset{N}{\underset{i=1}{\Sigma }}n_{i}d_{i}^{2}}$$

(2) $$PDI=\frac{d_{43}}{\frac{\overset{N}{\underset{i=1}{\Sigma }}d_{i}}{N}}$$

(3) $$d_{43}=\frac{\overset{N}{\underset{i=1}{\Sigma }}n_{i}d_{i}^{4}}{\overset{N}{\underset{i=1}{\Sigma }}n_{i}d_{i}^{3}}$$

where *d_i_* is the droplet diameter, *N* is the total number of droplets, and *n_i_* is the number of droplets having a diameter *d_i_*.

### 2.4. Rheological Measurements

Rheological measurements were carried out using a Physica MCR 501 rotational rheometer (Anton Paar, Graz, Austria) in continuous and oscillatory flow at 20 °C. Continuous flow measurements were performed in the shear rate range from 0.01 s^−1^ to 1000 s^−1^ using the cone-plate system (diameter 60 mm, angle 2°, gap 0.253 mm). The oscillatory measurements were carried out in the plate–plate system in the range of oscillation frequency from 0.01 rad/s to 100 rad/s. The plate diameter was 59.972 mm, and the gap width was 1 mm.

The oscillatory measurements were made in the range of linear viscoelasticity with the value of the strain amplitude equal to 1%. The range of linear viscoelasticity was determined by measuring the values of the G′ and G″ modules at increasing strain amplitude from 0.01% to 1000% and a constant frequency of 1 Hz. Rheological measurements were performed 48 h after the preparation of the emulsion.

### 2.5. Surface Tension

Surface tension measurements of the solutions and interfacial tension between the oil and continuous phase were conducted using a K12 tensiometer (Krüss GmbH, Hamburg, Germany). The du Nouy ring method was used, in which a platinum–iridium ring is immersed in the liquid and then pulled up at a constant speed (0.1 m s^−1^) until it detaches from the sample. Each measurement was repeated three times, and the surface and interfacial tension values are summarized in Table 4 and Table 5.

### 2.6. Statistical Analysis

To evaluate the significance of temporal changes in droplet diameters, statistical analysis was conducted using Statistica software, version 13.3 (TIBCO Software Inc., Santa Clara, CA, USA). The post hoc Duncan test was employed to determine which means exhibited statistically significant differences. All hypotheses were tested at a 0.05 significance level.

## 3. Results and Discussion

### 3.1. Stability of Emulsions

During the preliminary testing phase, the stability of the emulsions was analyzed by observing their samples placed in transparent containers after 2, 15, and 45 days, respectively. The emulsion that exhibited a separated oil phase was considered broken. The continuous phase of the emulsion consisted of distilled water and a 70% vol. ethyl alcohol solution containing 0.5% wt. to 2.0% wt. of HEC and MC and 0.3% wt. to 0.7% wt. of HPMC (Table 6). Stability analysis was also performed for emulsions where the continuous phase contained 0.5% wt. HPMC and EtOH concentrations of 20% vol. and 45% vol.

In all emulsions where the continuous phase did not contain ethyl alcohol, a separated oil phase was observed just two days after preparation. At the same time, no changes in the appearance of the emulsions containing ethyl alcohol were noted. After 15 days of preparation, phase separation was observed in emulsions containing 20% vol. and 45% vol. EtOH with 0.5% wt. HPMC. Among the emulsions with 70% vol. ethanol, phase separation occurred in those containing HEC at concentrations of 0.5% wt. and 0.8% wt., as well as MC at a concentration of 0.5% wt. The emulsions with added HPMC/EtOH remained stable throughout the entire observation period, except for the emulsion containing the lowest polymer concentration of 0.3% wt. and canola oil. In the case of the latter emulsion, separation of the oil phase could be observed after 45 days. After 45 days, emulsions with added MC at a concentration of 0.8% wt. also showed separation of both canola oil and mineral oil.

**Figure 1:**
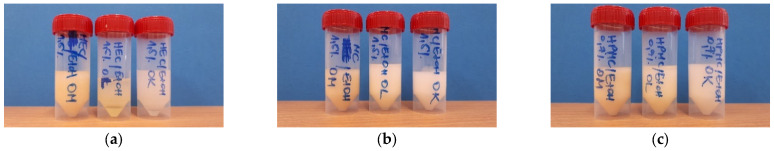
Sample photos of the emulsion taken 45 days after preparation: (**a**) HEC 1.5% wt./EtOH, (**b**) MC 1.5% wt./EtOH, (**c**) HPMC 0.7% wt./EtOH.

Based on preliminary measurements, emulsions showing no oil phase separation 45 days post-preparation were selected for further tests. These are emulsions in which the continuous phase contained 1.5% wt. of MC and HEC and 0.7% wt. of HPMC (Figure 1).

Emulsions containing at least 0.7% wt. of HPMC and 1.5% wt. of MC or HEC did not break during 45 days of storage. However, this does not imply that their properties remained unchanged, particularly regarding the size of the oil phase droplets. To assess any potential changes, the diameters of the emulsion droplets were measured using photographs taken under an optical microscope. For photographic documentation, emulsion samples were collected from various heights in the emulsion storage containers. Measurements were conducted on 10 independently collected fluid samples.

Approximately 20,000 droplet diameters were determined for each sample. Table 2 presents the calculated values of the Sauter diameter d_32_ for the developed emulsions, based on the droplet diameters measured immediately after emulsion preparation, as well as 30 and 5 months post-preparation.

Based on Ducan’s test, it was determined that the droplet diameters of the emulsions containing canola oil and linseed oil as the dispersed phase, with HEC/EtOH in the continuous phase, were significantly larger after 30 days compared to the diameters measured immediately after the emulsions were prepared. All emulsions containing MC/EtOH and HPMC/EtOH showed significantly larger droplet sizes 5 months after emulsion preparation. The supplementary data also include droplet size distributions obtained immediately after emulsion preparation and five months later (Figure S4). These distributions indicate that, over time, the proportion of larger droplets increases. The results indicate that the process of increase in droplet diameter is progressing, which is probably due to the phenomenon of Ostwald ripening. These findings align qualitatively with the observations for emulsions with added EtOH reported by Zeeb et al. [31] and Dickinson & Golding [32]. Table 3 additionally includes values for the polydispersity index (PDI). PDI values do not change noticeably over time. Statistically significant increases in PDI were observed only in emulsions with the addition of HEC and MC after 5 months.

Emulsion stability tests indicate that an addition of EtOH significantly decelerates the emulsion aging process. As mentioned earlier, emulsions without EtOH addition broke within just two days after preparation. In contrast, emulsions with added EtOH remained stable without separation even 5 months post-preparation, although their droplet diameters continued to increase over time. Sun et al. [30] obtained similar results, showing that emulsions with an addition of ethanol at a concentration of 90% vol. remained stable even after 45 days of preparation. The improved stability of emulsions with EtOH addition might be attributed to changes in the density and viscosity of the continuous phase, as well as alterations in interfacial tension.

Table 4 summarizes the density values of 70% vol. EtOH solution and the oils used in the tests. The density of the ethyl alcohol solution is significantly lower than that of water and close to the density of the oils used in the tests. The densities of the mineral oil and the ethanol solution differ by only 7 kg/m^3^. Reducing the density difference between the oil and water phases slows down sedimentation and creaming processes in emulsions.

Interfacial tension measurements were also performed for the oils used in the study and the 70% vol. EtOH solution (Table 5). Interfacial tension measurements involving HEC/EtOH, MC/EtOH, and HPMC/EtOH solutions were found unfeasible due to the extremely high viscosity of these polymer solutions.

The data in Table 5 indicate that the addition of EtOH reduced the interfacial tension at both the EtOH solution/canola oil and EtOH solution/linseed oil interfaces, though the changes were modest (by comparison, the addition of low-molecular-weight surfactants lowered the interfacial tension to around 5 mN/m). The interfacial tension values of the EtOH solution/mineral oil and water/mineral oil systems are identical. These results demonstrate that while the reduction of interfacial tension induced by the addition of EtOH may promote emulsion stabilization, it will not be the decisive factor for a rapid increase in the stability of emulsions containing EtOH.

**Figure 2:**
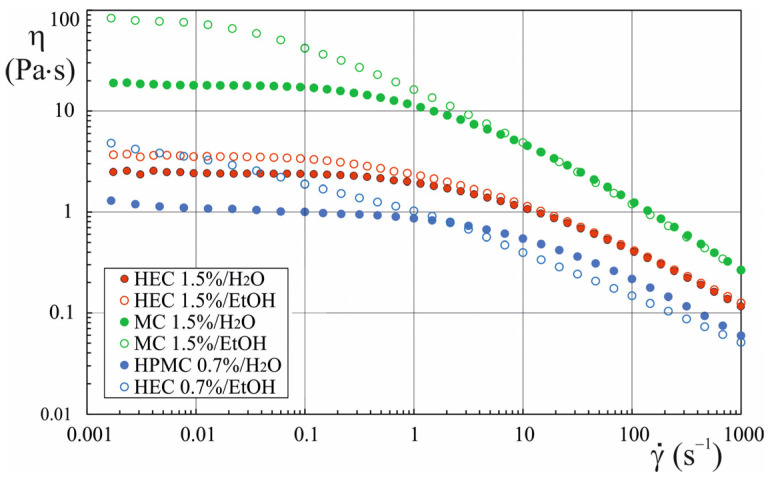
Viscosity curves for aqueous polymer solutions and polymers in water/ethanol mixtures (70% vol. EtOH).

The improved stability of emulsions with ethyl alcohol can be attributed to changes in the rheological properties of the continuous phase. The flow curves depicted in Figure 2 illustrate that the viscosity of HEC, HPMC, and MC solutions in a 70% vol. ethyl alcohol solution is significantly higher in the low shear rate range compared to the viscosity of aqueous solutions of these polymers. This trend was observed for all tested polymer concentrations (Figures S1–S3). Table 7 presents the zero viscosity values determined using the Cross model:

(4) $$\eta \left(\dot{\gamma }\right)=\eta_{\infty }+\frac{\eta_{0}-\eta_{\infty }}{1+{\left(\tau_{C}\cdot \dot{\gamma }\right)}^{n}}$$

where *η*_0,C_ and *η*_∞_ are the limiting viscosities at zero and infinite shear rates, respectively, *τ_C_* is the shear relaxation time, and *n* is an appropriate exponent.

### 3.2. Oscillatory Measurements

**Figure 3:**
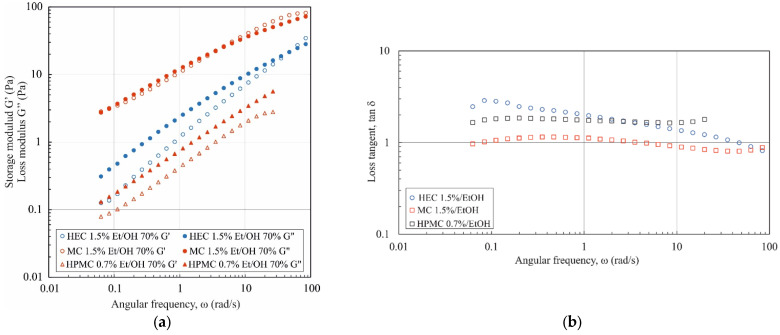
G′ and G″ moduli (**a**) and tan δ = G″/G′ (**b**) as a function of oscillation frequency ω for polymers in water/ethanol mixtures.

Figure 3 shows the mechanical spectra and the relationship between the phase shift angle tan δ = G″/G′ and the angular frequency for polymer solutions dissolved in a water/EtOH mixture.

In all cases, the values of the G′ and G″ moduli increased with angular frequency, with the highest values recorded for the 2% wt. methyl cellulose solution. For the 1.5% wt. HPMC and HEC solution, the G″ modulus is greater than the G′ modulus across the entire ω range, signifying a dominance of viscous over elastic properties. For 2% wt. HEC and 1.5% wt. MC solutions, the values of the G′ and G″ moduli intersect at high ω values. However, for the MC solution at a concentration of 2% wt., G′ consistently exceeds G″ across the entire angular frequency range (tan δ < 1). The mechanical spectra obtained are characteristic of semi-dilute polymer solutions.

**Figure 4:**
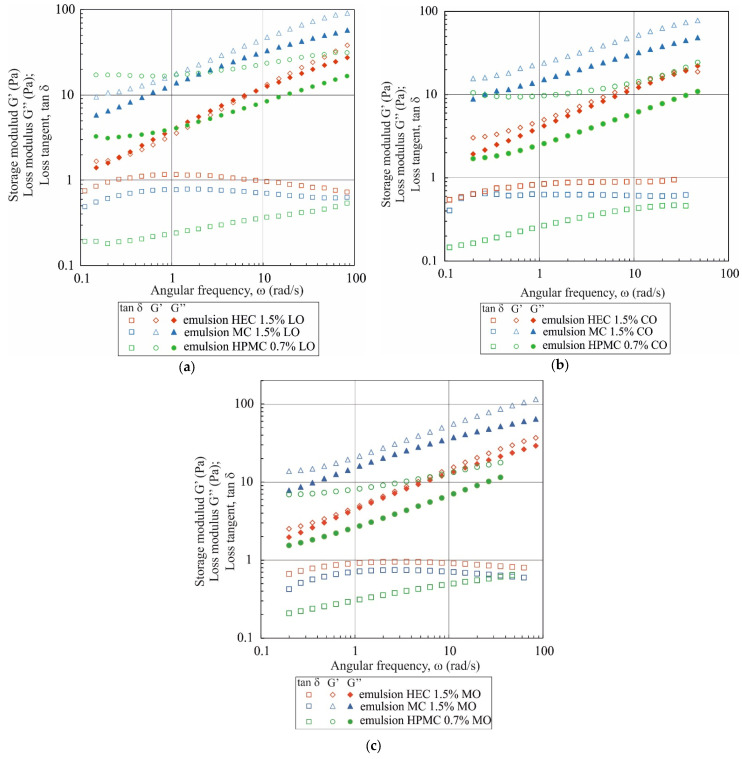
G′ and G″ moduli and tan δ = G″/G′ as a function of oscillation frequency ω for emulsions with (**a**) linseed oil, (**b**) canola oil, (**c**) mineral oil.

The mechanical spectra of emulsions (Figure 4) exhibit a different shape compared to those of polymer solutions in a water/EtOH mixture discussed previously. Only for the emulsion in which the continuous phase contained 1.5% wt. of HEC and linseed oil did the tan δ values exceed 1 within a specific range of oscillation frequencies. For all other emulsions, the G′ modulus values remained higher than the G″ modulus values across the entire range of angular frequency variations (tan δ < 1). For emulsions containing MC and HEC, the G′ = f(ω) and G″ = f(ω) curves run nearly parallel, with tan δ < 1 values being relatively large (ranging from approximately 0.75 to 0.95). This shape of the mechanical spectra is characteristic of what is termed ‘weak gels’.

**Figure 5:**
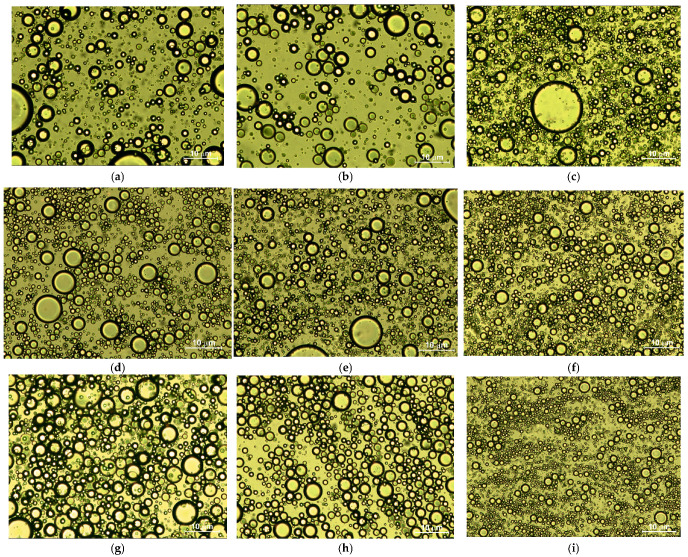
Sample photos of emulsions with added polymers/EtOH mixtures and various oils after preparation: (**a**) HEC/EtOH—canola oil, HEC 1.5% wt., (**b**) HEC/EtOH—linseed oil, HEC 1.5% wt., (**c**) HEC/EtOH—mineral oil, HEC 1.5% wt., (**d**) MC/EtOH—canola oil, MC 1.5% wt., (**e**) MC/EtOH—linseed oil, MC 1.5% wt., (**f**) MC/EtOH—mineral oil, MC 1.5% wt., (**g**) HPMC/EtOH—canola oil, HPMC 0.7% wt., (**h**) HPMC/EtOH—linseed oil, HPMC 0.7% wt., (**i**) HPMC/EtOH—mineral oil, HPMC 0.7% wt.

A different pattern of mechanical spectra was obtained for emulsions with added HPMC. In emulsions containing this polymer, a plateau in the storage modulus G′ appears at low oscillation frequencies, with tan δ values reaching approximately 0.17 within this range. Such low tan δ values are comparable to those observed in gels. Photographs in Figure 5 show that the emulsions obtained are flocculated, which directly leads to an increase in the values of the G′ and G″ moduli and a decrease in the tan δ value.

Two primary mechanisms of emulsion flocculation by polymers are described in the literature: depletion flocculation and bridging flocculation [40,41,42,43,44,45]. HPMC, MC, and HEC do not adsorb onto the surface of oil droplets; thus, flocculation of the emulsions used in the tests is likely due to the depletion effect [40]. Quantitative differences in G′ and G″ values for emulsions containing the same polymer but different oils are difficult to explain through photographs alone, as these emulsions not only vary in droplet diameter but are also highly polydisperse. Nevertheless, the shapes of the G′ = f(ω) and G″ = f(ω) curves for emulsions containing the same polymer but different oils are similar.

**Figure 6:**
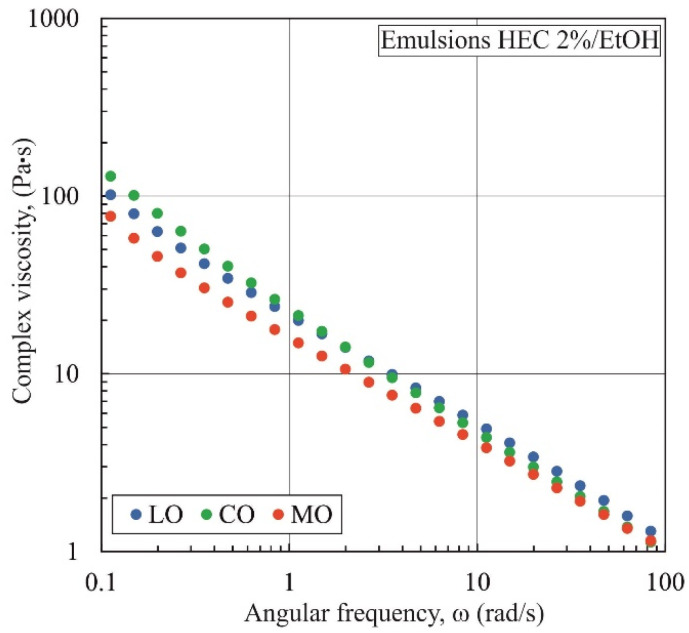
Comparison of the dependence of complex viscosity on oscillation frequency for emulsions containing linseed oil (LO), canola oil (CO), and mineral oil (MO) (emulsions with the addition of HEC (2% wt.) and EtOH (70% vol.)).

Figure 6 additionally shows the relationship between complex viscosity and oscillation frequency for emulsions containing 2% wt. HEC and various oils. The differences in complex viscosity among the emulsions are relatively small. The complex viscosity of the emulsion containing mineral oil is slightly lower, which may be attributed to differences in droplet size and droplet size distribution of the oil.

## 4. Conclusions

This paper presents findings on the stability and rheological properties of emulsions with a continuous phase containing one of three polysaccharides (HEC, MC, and HPMC) and EtOH (70% vol.). We have shown that with an appropriate concentration of ethanol in the continuous phase, relatively stable emulsions can be obtained without the addition of low-molecular-weight surfactant. The addition of EtOH to the emulsion rapidly slowed down the processes of emulsion sedimentation and creaming, as well as droplet coalescence. Rheological studies show that the addition of ethanol to the solutions of cellulose derivatives used in the tests causes a significant increase in zero viscosity. The increased emulsion stability is primarily due to the reduced density and elevated zero viscosity of the continuous phase. The resulting emulsions were viscoelastic fluids, which can be attributed to the aggregation of droplets. Adding methylcellulose to the continuous phase resulted in a particularly highly flocculated emulsion. Both rheological tests and photographs taken using an optical microscope revealed that a spatial network of small droplets had formed within the emulsion. The developed emulsions with viscoelastic properties can be used in the cosmetics and pharmaceutical industries as carriers of active substances (Active Ingredients or Active Pharmaceutical Ingredients).

## Figures and Tables

**Table 1:** Concentrations of individual compounds.

Polymer (% wt.)		Oil (% vol.)		Ethanol (% vol.)
HPMC	0.3	linseed mineral canola	20	70
	0.5			
	0.7			
HEC	0.2	linseed mineral canola	20	70
	0.5			
	0.8			
	1.5			
	2.0			
MC	0.2	linseed mineral canola	20	70
	0.5			
	0.8			
	1.5			
	2.0			

The given polymer and ethanol concentrations refer to the continuous phase.

**Table 2:** Change of droplet diameters *d*_32_ over time for the analyzed emulsions with polymers/EtOH mixtures (70% vol. EtOH).

Polymer/EtOH Emulsion	Cp (% wt.)	After Preparation	After 1 Month	After 5 Month
*d*_32_ (μm)				
Canola oil				
HEC	1.5	3.8	10.2 *	9.4 *
MC	1.5	8.7	9.5	17.1 *
HPMC	0.7	11.4	9.5	16.1
Linseed oil				
HEC	1.5	6.6	14.3 *	12.4 *
MC	1.5	7.2	8.4	15.7 *
HPMC	0.7	10.8	10.8	12.5 *
Mineral oil				
HEC	1.5	6.2	5.4	9.6 *
MC	1.5	5.8	6.0	7.4 *
HPMC	0.7	5.1	6.8	9.3 *

Diameter values marked with “*” were significantly different from the droplet diameters reported in the “After preparation” column in Duncan’s multiple range test (*p* < 0.05).

**Table 3:** Polydispersity index (PDI).

Polymer/EtOH Emulsion	Cp (% wt.)	After Preparation	After 1 Month	After 5 Months
Canola oil				
HEC	1.5	3.4	5.0 *	4.5 *
MC	1.5	4.5	3.6	5.9 *
HPMC	0.7	4.9	3.9	4.1
Linseed oil				
HEC	1.5	5.7	5.5	8.8 *
MC	1.5	3.6	4.1	5.6 *
HPMC	0.7	3.9	4.5	3.4
Mineral oil				
HEC	1.5	4.1	3.3	4.1
MC	1.5	4.0	3.4	3.2
HPMC	0.7	2.7	3.3	3.4

Polydispersity index marked with “*” was significantly different from the polydispersity index reported in the “After preparation” column in Duncan’s multiple range test (*p* < 0.05).

**Table 4:** Surface tension, density, and viscosity of oils and ethanol at 70% vol.

		Density (kg/m^3^)	Viscosity (Pa·s)	Surface Tension (mN/m)
oil	linseed	924	0.047	36.8
	canola	913	0.072	36.3
	mineral	860	0.044	33.5
aqueous ethanol solution	70% vol.	867	0.00255	29.9

**Table 5:** Interfacial tensions of H_2_O/oil and 70% EtOH/oil.

Interfacial Tension (mN/m)			
System	Linseed	Canola	Mineral
Water/oil	34.4	36.3	30.6
EtOH 70%/oil	27.6	28.4	30.6

**Table 6:** Emulsion stability after 15 and 45 days (70% vol. EtOH).

Emulsion	Cp (% wt.)	Canola Oil		Linseed Oil		Mineral Oil	
		15 Days	45 Days	15 Days	45 Days	15 Days	45 Days
HEC/EtOH	0.5	−	−	−	−	−	−
	0.8	−	−	−	−	−	−
	1.5	+	+	+	+	+	+
	2.0	+	+	+	+	+	+
MC/EtOH	0.5	−	−	+	−	−	−
	0.8	+	−	+	+	+	−
	1.5	+	+	+	+	+	+
	2.0	+	+	+	+	+	+
HPMC/EtOH	0.3	+	−	+	+	+	+
	0.5	+	+	+	+	+	+
	0.7	+	+	+	+	+	+

“+” homogeneous emulsion; “−” separated oil phase.

**Table 7:** Cross-model parameters.

Solution	*η*_0_ (Pa·s)	*n*	*τ_C_* (s)	*R* ^2^
HEC 1.5%/H_2_O	2.524	0.615	0.147	0.999
HEC 1.5%/EtOH	3.834	0.559	0.422	0.999
MC 1.5%/H_2_O	18.946	0.676	0.504	0.999
MC 1.5%/EtOH	92.314	0.613	11.918	0.998
HPMC 0.7%/H_2_O	1.170	0.583	0.135	0.999
HPMC 0.7%/EtOH	4.822	0.447	3.571	0.997

## Data Availability

The original contributions presented in the study are included in the article/Supplementary Materials; further inquiries can be directed to the corresponding author.

## Supplementary Materials

The following supporting information can be downloaded at: https://www.mdpi.com/article/10.3390/ma17246090/s1, Figures S1–S4, (Figure S1. Viscosity curves for aqueous MC solutions and MC in water/ethanol mixtures; Figure S2. Viscosity curves for aqueous HEC solutions and HEC in water/ethanol mixtures, Figure S3. Viscosity curves for aqueous HPMC solutions and HPMC in water/ethanol mixtures, Figure S4. Comparison of droplet distributions for fresh emulsions and 5 months after preparation: (a) HEC/EtOH after preparation, (b) HEC/EtOH after 5 months, (c) HPMC/EtOH after preparation, (d) HPMC/EtOH after 5 months, (e) MC/EtOH after preparation, (f) MC/EtOH after 5 months) and Table S1. The composition of oils used in the tests given by the manufacturers.

## References

[1] Mohanty A.K., Misra M., Drzal L.T. (2005). Natural Fibers, Biopolimers and Biocomposites.

[2] Abdulkhani A., Sousefi M.D., Ashori A., Ebrahimi G. (2016). Preparation and Characterization of Sodium Carboxymethyl Cellulose/Silk Fibroin/Graphene Oxide Nanocomposite Films. Polym. Test..

[3] Hou F., Fan L., Ma X., Wang D., Wang W., Ding T., Ye X., Liu D. (2018). Degradation of Carboxymethylcellulose Using Ultrasound and Glucanase: Pathways, Kinetics and Hydrolysates’ Properties. Carbohydr. Polym..

[4] Santa-Comba A., Pereira A., Lemos R., Santos D., Amarante J., Pinto M., Tavares P., Bahia F. (2001). Evaluation of Carboxymethylcellulose, Hydroxypropylmethylcellulose, and Aluminum Hydroxide as Potential Carriers for RhBMP-2. J. Biomed. Mater. Res..

[5] Yadollahi M., Gholamali I., Namazi H., Aghazadeh M. (2015). Synthesis and Characterization of Antibacterial Carboxymethyl Cellulose/ZnO Nanocomposite Hydrogels. Int. J. Biol. Macromol..

[6] Günter E., Popeyko O., Vitayazev F., Popov S. (2024). Effect of Callus Cell Immobilization on the Textural and Rheological Properties, Loading, and Releasing of Grape Seed Extract from Pectin Hydrogels. Gels.

[7] van der Linden H.J., Herber S., Olthuis W., Bergveld P. (2003). Stimulus-sensitive hydrogels and their applications in chemical (micro)analysis. Analyst.

[8] Ijaz F., Tahir H.M., Ali S., Ali A., Muzamil A., Manzoor H.H., Qayyum K.A. (2023). Biomolecules based hydrogels and their potential biomedical applications: A comprehensive review. Int. J. Biol. Macromol..

[9] Berardi A., Perinelli D.R., Merchant H.A., Bisharat L., Basheti I.A., Bonacucina G., Cespi M., Palmieri G.F. (2020). Hand sanitisers amid CoViD-19: A critical review of alcohol-based products on the market and formulation approaches to respond to increasing demand. Int. J. Pharm..

[10] Perinelli D., Berardi A., Bisharat L., Cambriani A., Ganzetti R., Bonacucina G., Cespi M., Giovanni F. (2021). Palmieri, Rheological properties of cellulosic thickeners in hydro-alcoholic media: The science behind the formulation of hand sanitizer gels. Int. J. Pharm..

[11] Villa C., Russo E. (2021). Hydrogels in Hand Sanitizers. Materials.

[12] Inada N., Maruyamaa R., Sawai H. Japanese Unexamined Patent Publication, 2010-126718. https://www.j-platpat.inpit.go.jp/c1801/PU/JP-2010-126718/11/en.

[13] Motoyama T., Katsuumi Y., Sasakura H., Nakamura T., Suzuki H., Tsuchiya K., Akamatsu M., Sakai K., Sakai H. (2022). Preparation of Highly Stable Oil-in-Water Emulsions with High Ethanol Content Using Polyglycerol Monofatty Acid Esters as Emulsifiers. J. Oleo Sci..

[14] Różańska S., Verbeke K., Różański J., Clasen C., Wagner P. (2019). Capillary Breakup Extensional Rheometry of Sodium Carboxymethylcellulose Solutions in Water and Propylene Glycol/Water Mixtures. J. Polym. Sci. B Polym. Phys..

[15] Komorowska P., Różańska S., Różański J. (2017). Effect of the Degree of Substitution on the Rheology of SodiumCarboxymethylcellulose Solutions in Propylene Glycol/Water Mixtures. Cellulose.

[16] Micklavzina L.B., Metaxas E.A., Dutcher S.C. (2020). Microfluidic Rheology of Methylcellulose Solutions in Hyperbolic Contractions and the Effect of Salt in Shear and Extensional Flows. Soft Matter.

[17] Vadodaria S.S., English R.J.J. (2016). Extensional rheometry of cellulose ether solutions: Flow instability. Cellulose.

[18] Martínez S., Espert M., Salvador A., Sanz T. (2022). The role of oil concentration on the rheological properties, microstructure, and in vitro digestion of cellulose ether emulsions. Food Hydrocoll..

[19] Brown A.F., Jones D.S., Woolfson A.D. (1998). The effect of alcoholic solvents on the rheological properties of gels composed of cellulose derivatives. J. Pharm. Pharmacol..

[20] Roberts M., Cespi M., Ford J.L., Dyas A.M., Downing J., Martini L.G., Crowley P.J. (2007). Influence of ethanol on aspirin release from hypromellose matrices. Int. J. Pharm..

[21] Yang Z., Huijuan Y., Hongshung Y. (2018). Characterisation of rheology and microstructures of κ-carrageenan in ethanol-water mixtures. Food Res. Int..

[22] Brunchi C.E., Morariu S., Bercea M. (2021). Impact of ethanol addition on the behaviour of xanthan gum in aqueous media. Food Hydrocoll..

[23] Zhang M., Zhou R., Han X., Wang J. (2024). The Fluorescence Property and Thermal Stability of SrAl_2_O_4_: Eu^2+^, Dy^3+^/Silicone Rubber Composites. J. Macromol. Sci. B.

[24] Wang J., Tang H., Han W., Han X. (2023). Thermal Degradation Kinetics of Alginate Fiber Containing a Novel Graphene-Based Flame Retardant. J. Macromol. Sci. B.

[25] McClements D.J. (2015). Food Emulsions: Principles, Practices, and Techniques.

[26] Medina-Torres L., Calderas F., Gallegos-Infante J.A., GonzalezLaredo R.F., Rocha-Guzman N. (2009). Stability of alcoholic emulsions containing different caseinates as a function of temperature and storage time. Colloids Surf..

[27] Kampf G. (2018). Efficacy of ethanol against viruses in hand disinfection. J. Hosp. Infect..

[28] Ferreira A.C., Winston S.S., Norton-Welch A.B. (2020). Influence of Ethanol on Emulsions Stabilized by Low Molecular Weight Surfactants. J Food Sci..

[29] Binks B.P., Fletcher P.D.I., Thompson M.A. (2013). Influence of propylene glycol on aqueous silica dispersions and particle-stabilized emulsions. Langmuir.

[30] Sun Y., Shen Y., Ding J., Ni X., Li C., Wang J., Yang C. (2022). High ethanol tolerance of oil-in-water Pickering emulsions stabilized by protein nanoparticles. Colloids Surf. A Physicochem. Eng..

[31] Dickinson E., Stainsby G. (1987). Progress in the formulation of food emulsions and foams. Food Technol..

[32] Dickinson E., Golding M. (1998). Influence of alcohol on stability of oil-in-water emulsions containing sodium caseinate. J. Coll. Interface Sci..

[33] Zeeb B., Gibis M., Fischer L., Weiss J. (2012). Influence of interfacial properties on Ostwald ripening in crosslinked multilayered oil-in-water emulsions. J. Coll. Interface Sci..

[34] Xu Q.Y., Nakajima M., Nabetani H., Iwamoto S., Liu X.Q. (2001). The effects of ethanol content and emulsifying agent concentration on the stability of vegetable oil-ethanol emulsions. J. Am. Oil Chem. Soc..

[35] Xi Z., Liu W., McClements D.J., Zou L. (2019). Rheological, structural, and microstructural properties of ethanol induced cold-set whey protein emulsion gels: Effect of oil content. Food Chem..

[36] Dickinson E. (2008). Interfacial structure and stability of food emulsions as affected by protein–polysaccharide interactions. Soft Matter.

[37] Burgaud I., Dickinson E. (1990). Emulsifying effects of food macromolecules in presence of ethanol. J. Food Sci..

[38] Li C., Li Y., Sun P., Yang C. (2013). Pickering emulsions stabilized by native starch granules. Colloids Surf..

[39] Leal-Castaňeda E.J., García-Tejeda Y., Hernández-Sánchez H., Alamilla-Beltrán L., Téllez-Medina D.I., Calderňn-Domínguez G., García H.S., Gutiérrez-Lňpez G.F. (2018). Pickering emulsions stabilized with native and lauroylated amaranth starch. Food Hydrocoll..

[40] Zhang X., Liu J. (2011). Effect of Arabic gum and xanthan gum on the stability of pesticide in water emulsion. J. Agric. Food Chem..

[41] Vélez G., Fernández M.A., Muñoz J. (2003). Role of hydrocolloids in the creaming of oil in water emulsions. J. Agric. Food Chem..

[42] Futamura T., Kawaguchi M. (2012). Characterization of paraffin oil emulsions stabilized by hydroxypropyl methylcellulose. J. Coll. Interface Sci..

[43] Dickinson E. (2009). Hydrocolloids as emulsifiers and emulsion stabilizers. Food Hydrocoll..

[44] Hogg R. (2013). Bridging flocculation by polymers. Kona Powder Part. J..

[45] Różańska S., Broniarz-Press L., Różański J., Mitkowski P.T., Ochowiak M., Woziwodzki S. (2013). Extensional viscosity of o/w emulsion stabilized by polysaccharides measured on the opposed-nozzle device. Food Hydrocoll..

