# Supplementary material for: Rheological Properties of Emulsions Stabilized by Cellulose Derivatives with the Addition of Ethyl Alcohol
Source: Materials (Basel). 2024 Dec 13;17(24):6090. doi: 10.3390/ma17246090 (PMC11677408; doi:10.3390/ma17246090)
Supplement: Supplementary file 1 [file materials-17-06090-s001.zip › materials-3335737-supplementary.pdf]

Supplementary Materials:

Rheological properties of emulsions stabilized with cellulose derivatives with the addition of ethyl alcohol

Sylwia Różańska, Jacek Różański, Patrycja Wagner, Ewelina Warmbier-Wytykowska

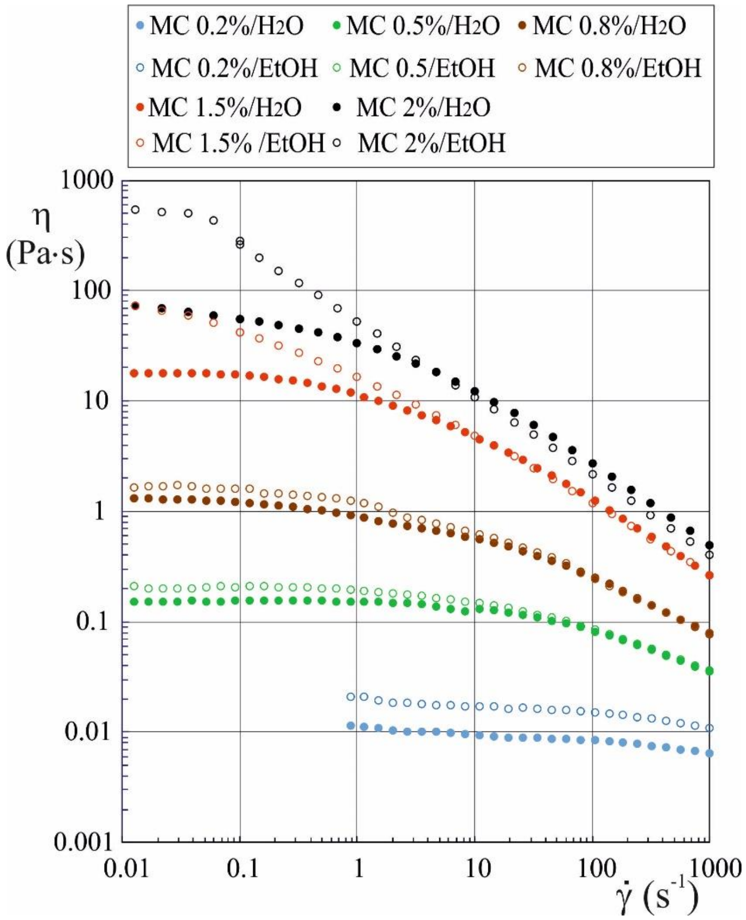

Figure S1. Viscosity curves for aqueous MC solutions and MC in water/ethanol mixtures

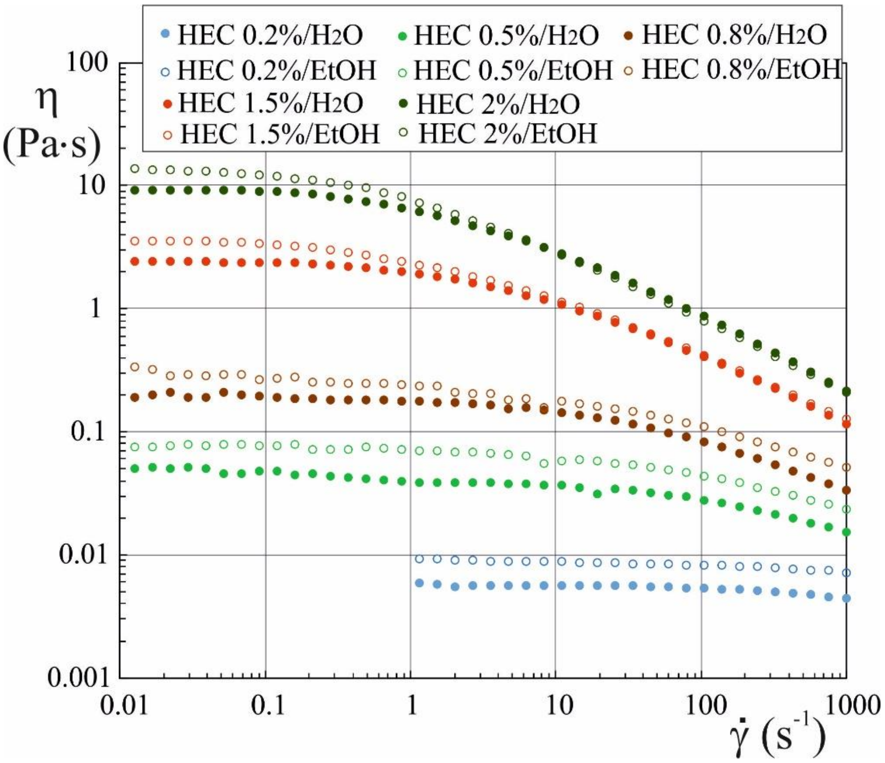

Figure S2. Viscosity curves for aqueous HEC solutions and HEC in water/ethanol mixtures

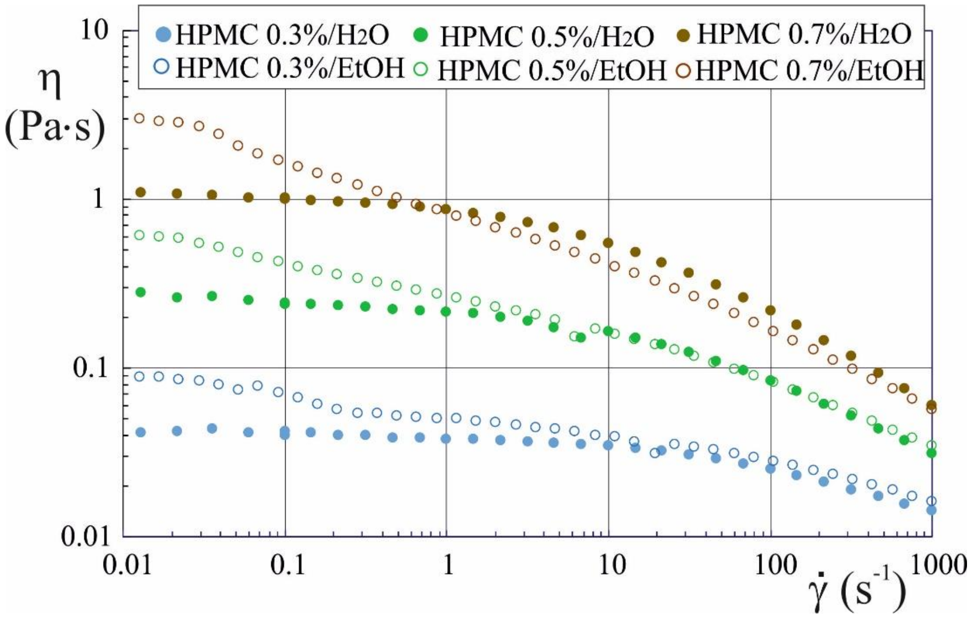

Figure S3. Viscosity curves for aqueous HPMC solutions and HPMC in water/ethanol mixtures

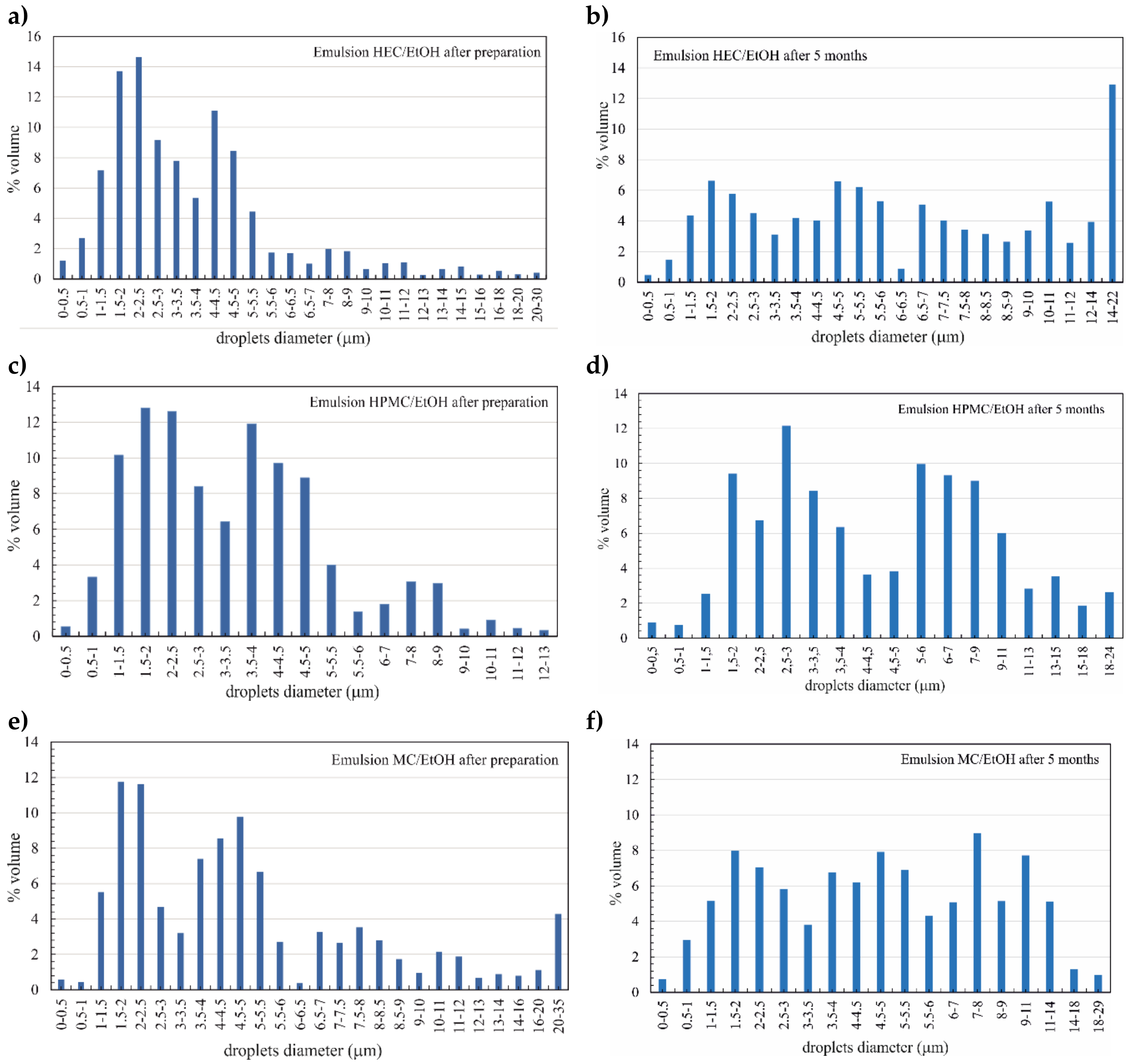

Figure S4. Comparison of droplet distributions for fresh emulsions and 5 months after preparation: a) HEC/EtOH after preparation, b) HEC/EtOH after 5 months, c) HPMC/EtOH after preparation, d) HPMC/EtOH after 5 months, e) MC/EtOH after preparation, f) MC/EtOH after 5 months.

Table S1. The composition of oils used in the tests given by the manufacturers

\	Canola	Linseed	Mineral
Fat, including:			mixture of liquid hydrocarbons.
Saturated acids	7.5 g	9.4 g	
Monounsaturated acids	65.5 g	17.3 g	
Polyunsaturated acids	26.5 g	73.1 g	
Vitamin E	25 mg	0 g	
Vitamin K	75 µg	0 g	
Omega acids 3	8.0 g	55.0 g	
